# Comparison of Two RapidArc Delivery Strategies in Stereotactic Body Radiotherapy of Peripheral Lung Cancer with Flattening Filter Free Beams

**DOI:** 10.1371/journal.pone.0127501

**Published:** 2015-07-01

**Authors:** Bao-Tian Huang, Jia-Yang Lu, Pei-Xian Lin, Jian-Zhou Chen, Yu Kuang, Chuang-Zhen Chen

**Affiliations:** 1 Department of Radiation Oncology, Cancer Hospital of Shantou University Medical College, Shantou, Guangdong, China; 2 Department of Nosocomial Infection Management, the Second Affiliated Hospital of Shantou University Medical College, Shantou, Guangdong, China; 3 Medical Physics Program, University of Nevada Las Vegas, Las Vegas, NV, 89154, Unites States of America; Institut Gustave Roussy, FRANCE

## Abstract

**Purpose:**

To investigate the performance of using partial arc (PA) and full arc with avoidance sectors (FAAS) in stereotactic body radiotherapy (SBRT) of peripheral lung cancer with flattening filter free (FFF) beams.

**Methods:**

Eighteen patients with primary (T1 or T2) non-small-cell lung cancer (NSCLC) or lung metastatic were selected for this study. Nine patients with a gross tumor volume (GTV) <= 10 cc were designated as the small tumor group. The other nine patients with a GTV between 10 cc and 44 cc were assigned to the large tumor group. The treatment plans were generated in eighteen patients using PA and FAAS techniques, respectively, and delivered with a Varian TrueBeam Linac. Dosimetry of the target and organs at risk (OARs), monitor unit (MU), out-of-field dose, and delivery time were statistically analyzed. Delta4 and portal dosimetry were employed to evaluate the delivery accuracy.

**Results:**

For the small tumor group, compared with the PA plans, the FAAS plans significantly achieved a lower MU/fraction, out-of-field dose and a shorter treatment time (*p*<0.05), but the target dose was slightly higher than that delivered by PA plans (*p*<0.05). For the large tumor group, the PA plans significantly attained a shorter treatment time (*p*<0.05), whereas MU/fraction, out-of-field dose and dose to OARs were comparable between the two plans (*p*>0.05). Furthermore, all plans generated from the eighteen patients achieved a high pass rate in patient-specific quality assurance, with all the gamma indices greater than 97% at the Γ_3mm, 3%_ threshold.

**Conclusion:**

This study suggests that the FAAS technique is more beneficial for the small tumor patients undergoing lung SBRT with FFF beams because of its higher treatment efficiency and MU reduction. However, for the large tumor patients, the PA technique is recommended due to its higher treatment efficiency.

## Introduction

Lung cancer remains the most frequent cause of death from cancer in both men and women worldwide [1, 2]. Clinical studies have indicated that stereotactic body radiation therapy (SBRT) is effective for both primary and metastatic lung cancer. To patients with medically inoperable early stage peripheral non-small-cell lung cancer (NSCLC), SBRT has achieved a favorably high local control rate, up to 88–92% [3].

More recently, RapidArc combined with flattening filter free (FFF) beams has become an extraordinarily attractive dose delivery technique in lung SBRT with high dose per fraction, which leads to a clinically meaningful reduction in treatment time, consequently improving patient stability and treatment accuracy during the course of lung cancer treatment [4–6].

SBRT with RapidArc and FFF beams involving one or more full arcs rotation strategy appears to be suboptimal for peripheral lung cancer as it increases the disadvantageous dose to the contralateral lung, which potentially increases the incidence of radiation induced pneumonitis (RIP) [7]. Therefore, partial arc (PA) and full arcs with avoidance sectors (FAAS) which could maintain lower pneumonitis rate in the contralateral lung are the most common used techniques in lung SBRT [8–11]. However, the dosimetric effect and treatment efficiency between the two delivery techniques remains unknown and needs further investigation.

In this study, we investigated, for the first time to the best of our knowledge, the dosimetric effects of two RapidArc delivery techniques, PA and FAAS, on SBRT in peripheral lung cancer with FFF beams. Dosimetric analysis was performed to determine which planning technique (PA *vs* FAAS) is optimal for MU, out-of-field dose reduction and the improvement of treatment efficiency according to different tumor sizes.

## Materials and Methods

### Ethics statement

The protocol was approved by the Ethics Committees of Cancer Hospital of Shantou University Medical College. Since this is not a treatment-based study, our institutional review board waived the need for written informed consent from the participants. But the patient information was anonymous to protect their confidentiality.

### Patient selection

Eighteen patients previously diagnosed with primary (T1 and T2) NSCLC or lung metastatic with single peripheral lesion no larger than 5 cm and treated with IMRT or RapidArc at Cancer Hospital of Shantou University Medical College were retrospectively selected for this study. All patients were chosen by a radiation oncologist with lung SBRT expertise to present different challenge levels for different tumor sizes and peripheral locations that had needed an optimal lung SBRT treatment strategy with RapidArc and FFF beams in the clinic.

According to the volume-adapted dosing strategy described below, based on different tumor volumes, the patients were separated into small and large tumor groups, respectively. Nine patients with a gross tumor volume (GTV) < = 10 cc were designated as the small tumor group [12]. The remaining nine patients with a GTV between 10 cc and 44 cc were assigned to the large tumor group.

### Immobilization and CT scanning

All patients were treated in supine position with arms crossed above their heads. A vacuum bag (Medtec Medical, Inc, Buffalo Grove, IL) or a thermoplastic mask (Guangzhou Klarity Medical & Equipment Co., Ltd, Guangzhou, China) was used to immobilize the thoracic regions. Of the eighteen patients, two patients were received contrast-enhanced CT scan followed by four-dimensional computed tomography (4DCT) scans using Brilliance CT with Big Bore (Cleveland, OH, USA). The remaining sixteen patients were only received the conventional contrast-enhanced CT scans. The contrast-enhanced CT thickness was set to 3 mm per slice. The CT images were then transferred to Eclipse treatment planning system (V10, Varian Medical System, Inc., Palo Alto, CA) for target volumes and organs at risk (OARs) delineation and treatment planning.

### Target and OARs delineation

For 4DCT images, the gross tumor volume (GTV) accounting for tumor motion on all ten phases of the 4DCT images were contoured within the CT pulmonary windows by one radiation oncologist with expertise in lung SBRT. The GTV of the ten phases were then combined to form internal target volume (ITV). To account for the set-up uncertainties and potential baseline tumor shift, the planning target volume (PTV) was created by adding a uniform 5 mm margin expansion from ITV.

For conventional contrast-enhanced CT images, the GTV was also contoured within the CT pulmonary windows and the PTV was created accounting for tumor motion under the guidance of fluoroscopic examination using a simulator.

OARs contouring include aorta, bronchial tree, esophagus, spinal cord, chest wall, heart, trachea and superior vena cava (SVC). The OARs were contoured by the same radiation oncologist according to the guidelines of the RTOG 0915 protocol [13].

### The volume-adapted dosing strategy

All plans were created on the contrast-enhanced CT images. A biologically effective dose (BED) of ≥ 100 Gy could achieve high rates of local control in SBRT for both primary and metastatic lung tumors [14]. The rate-limiting factor for local control is tumor volume, with the evidence that eleven-month locate control was 93–100% for tumors up to 12 cc but only 47% for tumors >12 cc with dose range of 15–30 Gy per fraction [12, 15]. Thus, a volume-adapted dosing strategy for lung tumor SBRT was used in this study.

For small tumor group, the patients were prescribed with 25 Gy in single fraction regimens with BED < 100 Gy. For the large tumor group, the patients were prescribed with 48 Gy in four factions according to RTOG 0915 protocol with BED > 100 Gy. The purpose of this dosing strategy used was to balance locate control and toxicities for patients with smaller tumors. The patients’ characteristics were summarized in Table 1.

**Table 1:** Detailed information of eighteen lung tumor patients investigated.

Patient	Age	D_PTV_ (cm)	FZ (cm)	FS (Gy)	Tumor Location
1	76	3.8	5.3×4.9	1×25	Left lower lobe (anterior)
2	77	3.1	4.2×3.8	1×25	Mid right lung adjacent to chest wall
3	74	3.3	5.6×5.0	1×25	Left upper lobe
4	73	2.9	4.4×4.2	1×25	Left lower lobe adjacent chest wall (posterior)
5	45	2.5	4.6×4.7	1×25	Mid right lung
6	57	2.8	4.6×4.1	1×25	Right upper lobe
7	33	2.6	4.7×4.5	1×25	Right lower lobe
8	53	3.2	4.9×4.7	1×25	Right upper lobe adjacent chest wall (posterior)
9	76	3.6	4.8×3.9	1×25	Mid left lung
10	70	4.4	6.0×6.0	4×12	Right upper lobe adjacent chest wall (posterior)
11	67	4.5	5.7×5.6	4×12	Left upper lobe (anterior)
12	48	6.0	9.3×8.7	4×12	Left upper lobe
13	58	5.6	8.4×8.6	4×12	Right upper lobe adjacent chest wall
14	76	6.2	8.7×7.1	4×12	Mid-posterior left lung
15	78	4.4	6.2×6.3	4×12	Right upper lobe adjacent chest wall
16	70	5.5	6.4×6.8	4×12	Right upper lobe adjacent chest wall (posterior)
17	67	5.0	7.0×6.8	4×12	Left upper lobe adjacent chest wall (posterior)
18	80	4.5	6.0×5.5	4×12	Left upper lobe adjacent chest wall

D_PTV_ = diameter of PTV in greatest dimension;

FZ = field size; FS = fraction scheme.

### Treatment planning

For all patients, two different treatment strategies, PA and FAAS, were used to implement the lung SBRT plans with FFF beams, respectively. The PA plans were generated through using two coplanar arcs which rotates from 179° to 320° (the stop angle is slightly different from patient to patient to prohibit the beams from entering contralateral lungs) clockwise and counterclockwise if targets locate at the left lung. The FAAS plans were generated through using two 360° coplanar arcs with avoidance sector to exclude entrance of beams through contralateral lungs. Avoidance sectors are ranges of gantry rotation where no MU are delivered (i.e., the beam is turned off in this avoidance sector areas). The mirror treatment strategies were also applied to the tumor on the right lung.

**Fig 1:**
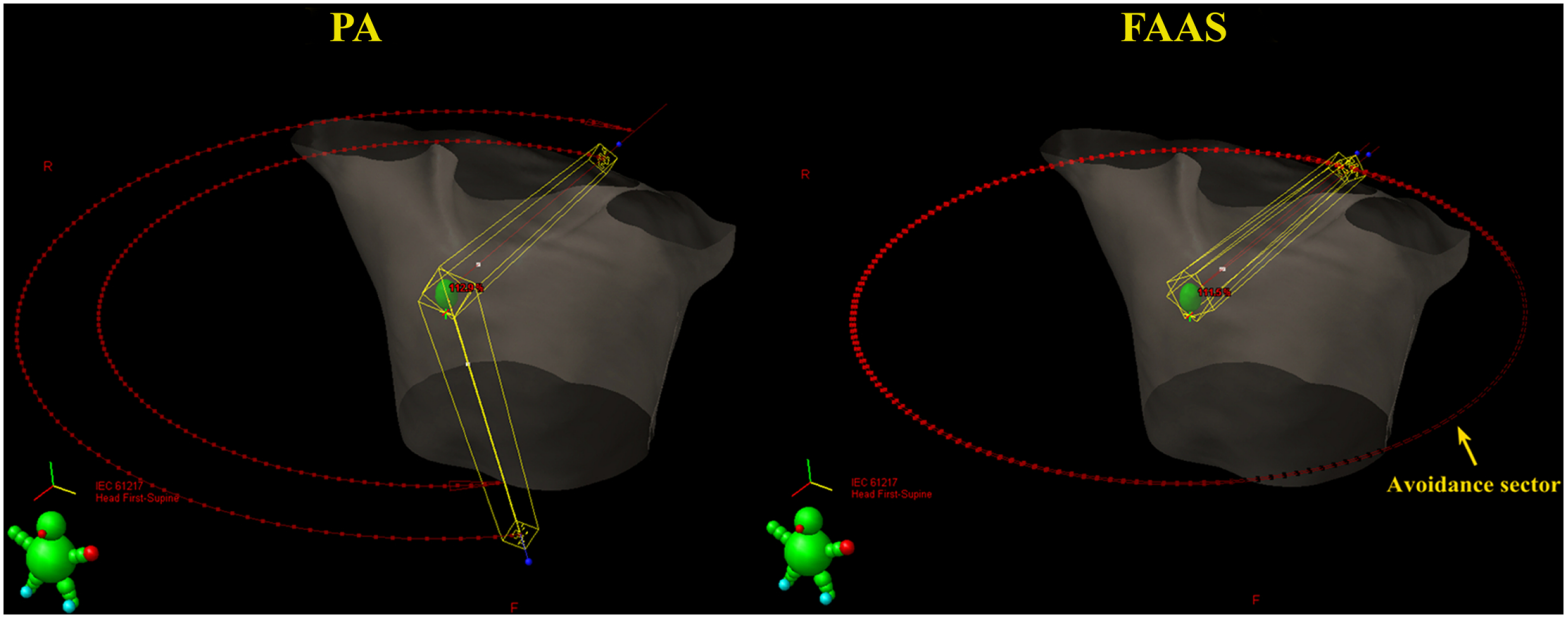
Schematic diagram of PA and FAAS. PA = partial arc; FAAS = full arc with avoidance sector.

The collimator settings were the same in both strategies. Collimator angles for all plans were set to 30° in one arc and the complementary angle 330° for the other. Schematic diagrams for the two types of arcs were shown in Fig 1. The grouped fields were aligned to the center of PTV. To ensure a steep dose fall-off outside the PTV, a 6 mm thick ring structure was created surrounding the target. The dose constraints for the target volume and different OARs followed the guidelines of the RTOG 0915 protocol [13].

The plans using PA or FAAS strategies were optimized using the same optimization constraints. During the process of the optimization, we utilized 114 and 178 controls points for PA and FAAS techniques, respectively. Dose calculations were carried out using the anisotropic analytical algorithm (AAA_10028) with a grid resolution of 2.5 mm, with the heterogeneity correction taking into account. The final dose calculation was normalized to ensure at least 95% of the PTV volume received the prescription dose. The 6 MV FFF photon beams was used for treatment and delivered by a TrueBeam Linac (Varian Medical Systems, Inc, Palo Alto, CA) equipped with a millennium multileaf collimator (MLC, spatial resolution of 5 mm at isocenter for the central 20 cm and 10 mm for the outer 20 cm). A maximum dose rate on the central beam axis of 1400 MU/min was employed in the optimization process. The plan calculated at the first time was used as a basedose plan for further optimization to compensate any underdose or “dose cloud” areas in the previously calculated plan by giving or reducing extra dose.

### Dosimetric analysis

Different dosimetric metrics were used to evaluate the dosimetric effects of PA and FAAS plans on SBRT in peripheral lung cancer with FFF beams.

D_98%_, D_2%_ and D_mean_ were used to evaluate PTV. D_98%_, D_2%_ represented the dose received by 98% and 2% of the target. D_mean_ represented the mean dose received by the target. Conformity index (CI) was used to compare the plan conformity in the two treatment strategies. CI_80%_, CI_60%_, CI_50%_ and CI_40%_ were defined as the volumes encompassed by the 80%, 60%, 50% and 40% isodose lines divided by the volumes of PTV encompassed by the same isodose levels, respectively [3].

The maximum dose and various dose parameters (V_x_) to specific OARs were generated for the plans to assess their effectiveness in OAR sparing. The maximum dose was used to evaluate the effectiveness of maintaining OARs’ sparing profiles by both treatment strategies in aorta, spinal cord, esophagus, heart, trachea, bronchial tree and SVC. In addition, four dosimetric metrics of lung V_5_, V_10_, V_20_, and mean lung dose (MLD) and three metrics of chest wall (V_45_, V_30_ and V_20_) were also included [3, 16].

### Peripheral doses outside the treatment fields

AAA was widely used in the dose calculation of treatment planning, but uncertainties also existed due to its accuracy for estimating the peripheral dose [17]. The method of choice for peripheral dose assessment is phantom measurements or Monte Carlo (MC) simulations. Peripheral dose cannot be easily calculated with a high degree of accuracy due to the restricted CT scans to the treated region, defective head-scatter models without taking the treatment head leakage into account and lack of models for deriving the peripheral dose from fluence information [18].

To compare peripheral doses outside the treatment field delivered by PA and FAAS plans, a thorax phantom (CIRS, Inc, Norfolk, VA) combined with a FC-65G ionization chamber (0.6 cm^3^) with buildup cap (Standard Imaging, Middleton, WI) was used to measure the ionization of the photon beams as a function of distance from the isocenter. The thorax phantom was constructed of tissue equivalent epoxy materials to simulate photon scattering effects in the patient during treatment. The thorax phantom with a tumor rod (3 cm in diameter) was placed at the isocenter and the ionization chamber was placed at 20, 40, and 60 cm away from it, respectively, to measure the out-of-field doses. Because the head leakage is the predominant contributor to the out-of-field dose at a distance far from the treatment field (> 15 cm) [19], the tip of the chamber was placed to face towards the gantry to ensure the accuracy of measurement. DOSE-1 electrometer (IBA, Munich, Germany) was used to record the measurement by connecting itself to the chamber using extension cable.

The absorbed dose was calculated as follows. The out-of-field dose was then converted to mGy/Gy for comparison.

*D*_*air*_ = *M*_*u*_×*N*_*x*_×0.876×*K*_*att*_×*K*_*m*_ Where *D*_*air*_ (cGy) was the absorbed dose in the air, *M*_*u*_ was the readout on the electrometer and *N*_*x*_ was the exposure calibration factor of an ionization chamber (equal to 1.033 in this study). 0.876 (cGy/R) was the coefficient of exposure to absorbed dose in the air. *K*_*att*_ was the correction factor of absorption and scattering of an ionization chamber, *K*_*m*_ was the factor to take account of non-air equivalence of the ionization chamber wall and buildup cap material (*K*_*att*_ *×K*_*m*_ was equal to 0.987).

### Treatment efficiency

To compare the treatment efficiency delivered by PA and FAAS plans, the treatment time for each plan was recorded by performing the dry-run function on the Linac. It was recorded from the start of the first arc and the end of the second arc, including the intervals between the two arcs and the gantry rotation time in the avoidance sectors. The actual measured treatment time was also cross-checked with the estimated one according to the empirical equations as follows:

For PA plan,

$$T=\frac{MU}{dose\;rate}+\frac{5}{60}$$

For FAAS plan,

$$T=\frac{MU}{dose\;rate}+\frac{5}{60}+\frac{(Avoidance\;\sec tor)\;{}^{\circ}}{6\times 60}$$

### Plan QA

Each plan was verified to assess the agreement between calculated and delivered doses using both 3D detector array delta4 (ScandiDos, Uppsala, Sweden) and electronic portal imaging devices (EPID) mounted on the TrueBeam Linac. For Delta4 measurement, we implemented 1069 p-type silicon diodes for gamma analysis in a 20 cm × 20 cm detection area. The spatial resolution was 5 mm for the central 6 cm × 6 cm area, and 10 mm for the outer area. The results were evaluated in terms of gamma index (*Γ*_3mm, 3%_), which is calculated using spatial and dosimetric limits of 3 mm distance-to-agreement and a 3% dose difference, respectively.

### Statistical analysis

All reported values are expressed as mean ± standard deviation of the mean. The data were compared using paired t-test when the data obey normal distributions; otherwise Wilcoxon signed-rank test was used. A *p*-value < 0.05 was regarded as statistically significant. All statistical analysis was performed in SPSS 19.0 (SPSS, Inc, Chicago, IL).

## Results

**Fig 2:**
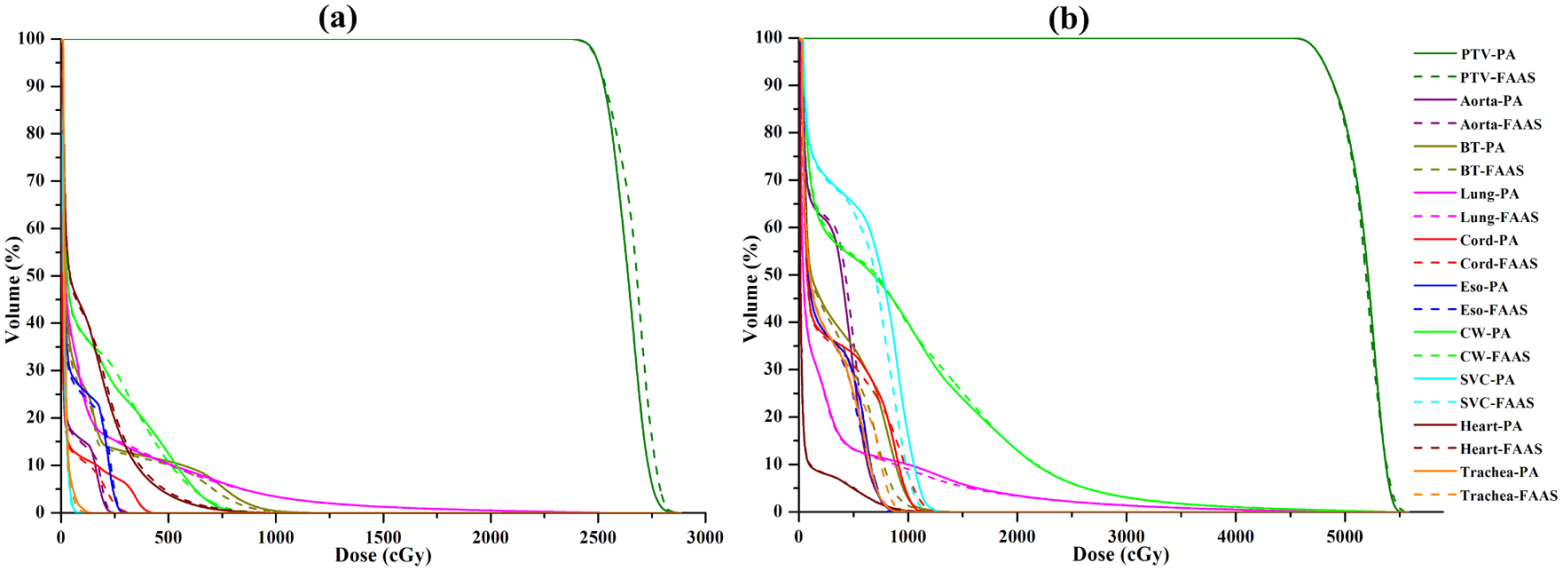
A representative DVH from the PA and FAAS plans in the two groups of patients. (a) small tumor group; (b) large tumor group. BT = bronchial tree; Eso = esophagus; CW = chest wall; SVC = superior vena cava.

**Fig 3:**
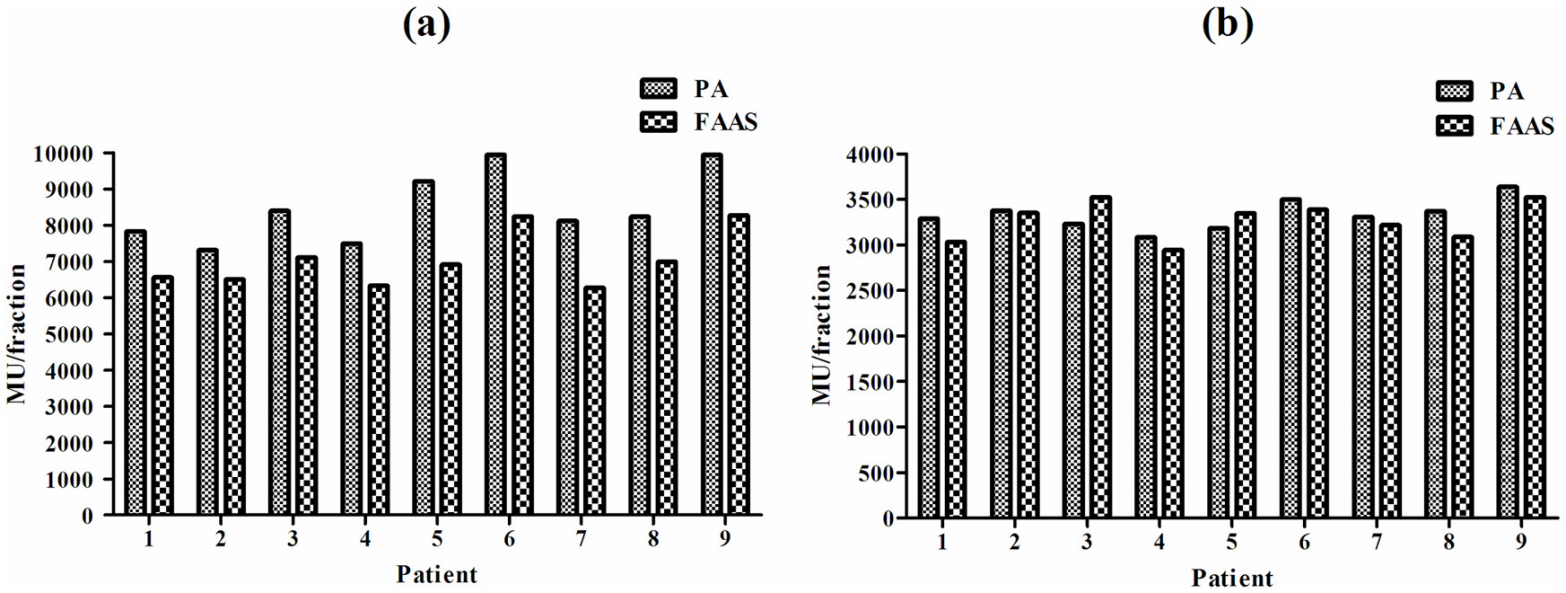
MU/fraction in the two groups of patients investigated. (a) small tumor group; (b) large tumor group.

The statistical analysis of dosimetric metrics comparison for PTV and different OARs in all patients were summarized in Table 2. All the plans met the dose constraints described in the RTOG 0915 protocol and achieved a similar level of PTV coverage. For the small tumor group, a higher D_2%_ and D_mean_ of PTV was observed in the FAAS plans (*p* < 0.05). The FAAS plans attained a lower maximum dose in aorta compared to the PA plans (*p* < 0.05). The conformity index CI_80%_ and CI_60%_ of FAAS plans were inferior to those of PA plans (*p* < 0.05). Above all, MU/fraction delivered by the FAAS plans were significantly reduced compared with those delivered by PA plans (*p* < 0.05). In the large tumor group, both FAAS and PA plans had a similar PTV and OARs dose. However, the conformity index CI_80%_ and CI_60%_ seem to be inferior to those of PA plans. Unlike the small tumor group, MU/fraction delivered by both plans was comparable. A representative dose-volume histogram (DVH) from the PA and FAAS plans in the small and large tumor groups are shown in Fig 2. MU/fraction from individual patient was displayed in Fig 3.

**Table 2:** Summary of PTV, OARs and MU/fraction for the small and large tumor patients using two techniques.

Metrics	Unit	Small tumor group			Large tumor group		
		PA	FAAS	*p*	PA	FAAS	*p*
PTV D_98%_	%	98.5±0.2	98.6±0.2	0.339	98.1±0.1	98.1±0.2	0.236
PTV D_2%_	%	111.4±1.1	112.4±1.8	0.011*	115.8±4.1	116.5±3.9	0.105
PTV D_mean_	%	105.9±0.6	106.5±1.0	0.007*	108.5±2.1	108.9±2.0	0.169
Aorta D_max_	Gy	4.0±2.3	3.7±2.1	0.008*	17.6±10.3	16.5±9.6	0.066
Bronchial tree D_max_	Gy	5.4±5.0	5.5±5.0	0.374	15.5±8.5	14.5±7.1	0.120
Chest wall V_45_	cc	0.0±0.0	0.0±0.0		4.4±4.6	4.3±4.5	0.431
Chest wall V_30_	cc	0.0±0.0	0.0±0.0		19.4±15.5	19.7±15.7	0.018*
Chest wall V_20_	cc	1.5±3.1	1.5±3.0	0.080	79.1±53.4	80.6±54.0	0.161
Spinal cord D_max_	Gy	3.4±1.3	3.5±1.4	0.783	9.9±3.4	9.8±3.3	0.708
Esophagus D_max_	Gy	3.0±1.3	3.1±1.2	0.617	10.3±2.9	10.1±3.0	0.588
Heart D_max_	Gy	6.2±7.7	5.9±7.9	0.182	10.3±6.8	9.7±6.0	0.240
Lung V_5_	%	5.3±2.3	5.2±2.4	0.523	13.3±3.6	13.5±3.6	0.214
Lung V_10_	%	1.9±0.8	1.9±0.8	0.452	9.3±3.3	8.9±2.9	0.110
Lung V_20_	%	0.4±0.2	0.4±0.3	0.332	3.9±1.8	3.9±1.8	0.753
Lung MLD	Gy	1.0±0.3	1.0±0.3	0.790	3.1±1.0	3.1±1.0	0.744
SVC D_max_	Gy	2.2±1.9	2.1±1.7	0.594	8.0±4.7	7.7±4.4	0.328
Trachea D_max_	Gy	1.1±1.4	1.1±1.5	0.310	7.9±2.9	7.7±2.9	0.219
CI_80%_		1.73±0.1	1.74±0.1	0.002*	1.5±0.0	1.5±0.0	0.033*
CI_60%_		3.0±0.2	3.0±0.2	0.038*	2.5±0.1	2.5±0.1	0.016*
CI_50%_		4.2±0.3	4.3±0.3	0.164	3.6±0.1	3.6±0.2	0.109
CI_40%_		6.8±0.5	6.8±0.5	0.619	5.8±0.3	5.8±0.3	0.260
MU/fraction		8500±986	7023±756	0.000*	3331±167	3268±211	0.342

PA = partial arc; FAAS = full arc with avoidance sector; PTV = planning target volume; MLD = mean lung dose; CI = conformity index; SVC = superior vena cava. Values are mean ± SD.

* stands for statistically significant.

**Fig 4:**
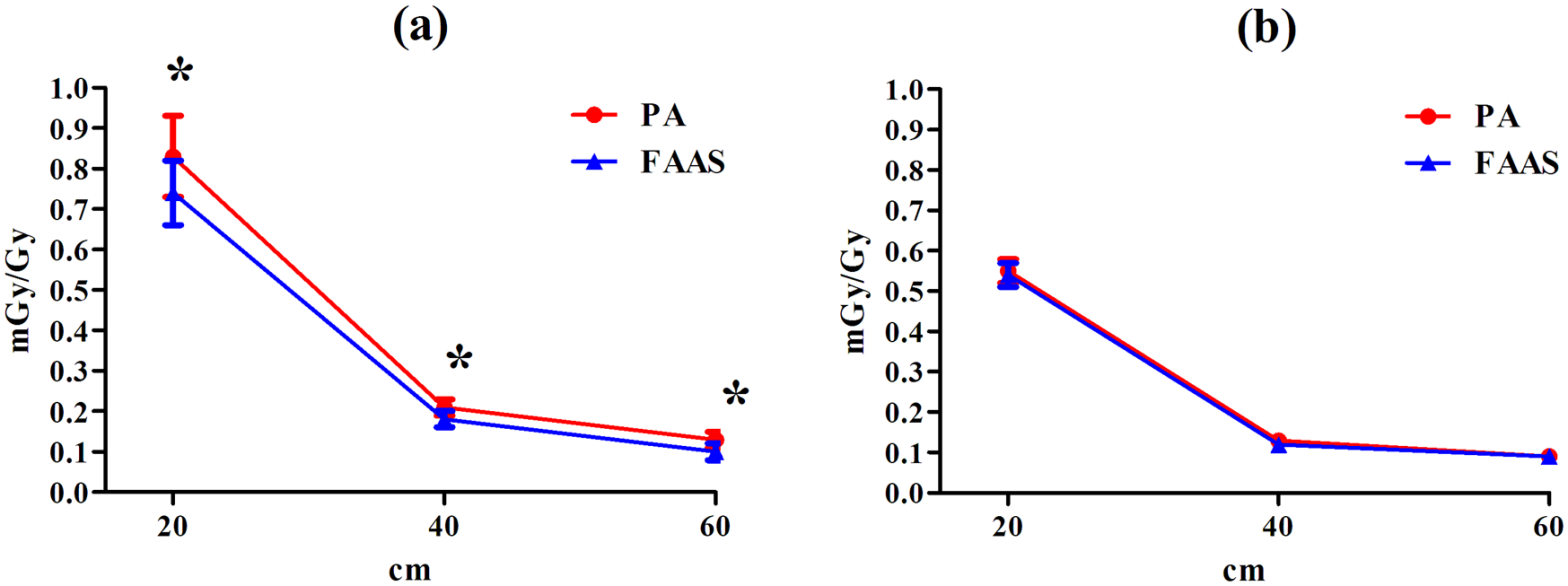
Out-of-field dose comparison in the two groups of patients. (a) small tumor group; (b) large tumor group. * stands for statistically significant.

The peripheral doses at lateral distance 20, 40 and 60 cm from the isocenter delivered by both plans were also evaluated in Fig 4. In the small tumor group, the FAAS plans show significantly reduced peripheral doses along the longitudinal direction from the isocenter than that contributed by the PA plans (*p <* 0.05). In contrast, in the large tumor group, no significant differences were observed in the peripheral doses delivered by both FAAS and PA plans (*p* > 0.05).

**Fig 5:**
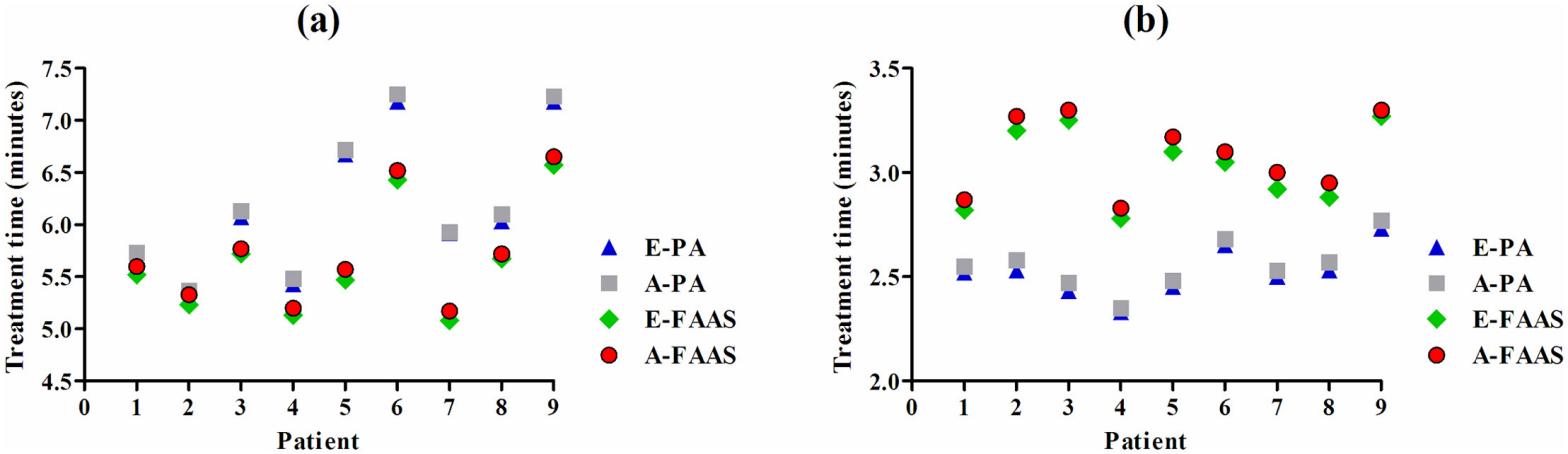
Treatment efficiency in PA and FAAS plans. (a) small tumor group; (b) large tumor group. E-PA = estimated treatment time of PA; A-PA = actual treatment time of PA; E-FAAS = estimated treatment time of FAAS; A-FAAS = actual treatment time of FAAS.

The treatment efficiency of both plans was also investigated via measuring the delivery time. It can be observed from Fig 5 that the estimated treatment times were in an excellent agreement with the measured ones, regardless of small tumor or large tumor group. The mean value of actual treatment time in the small tumor group was 6.2±0.7 minutes for the PA plans and only 5.7±0.5 for the FAAS plans (*p* < 0.05). In contrast, the PA plans attained a shorter treatment time compared to the FAAS plans (2.6±0.1 *vs* 3.1±0.2 minutes on average, *p* < 0.05) in the large tumor group.

Table 3 summarized the γ analysis for both plans using delta4 and portal dosimetry. Both verification techniques show very high agreement between calculated doses and measured doses. Less than 2% of the analyzed areas exceeded γ value > 1. Meanwhile, the maximum γ or mean γ value showed similar results for both verification techniques.

**Table 3:** Plan verification results averaging from eighteen patients.

	Delta4			Portal dosimetry		
	γ<1, %	Maximum γ	Mean γ	γ<1, %	Maximum γ	Mean γ
PA	99.50±0.37	1.35±0.62	0.18±0.12	99.45±0.34	1.27±0.29	0.26±0.14
FAAS	99.20±0.42	1.24±0.23	0.20±0.09	99.30±0.26	1.19±0.33	0.32±0.18

Data presented as mean ± standard deviation. PA = partial arc; FAAS = full arc with avoidance sector.

## Discussion

In this study, we found that the MU/fraction delivered by FAAS plans in the small tumor group were significantly reduced than that delivered by PA plans. Consequently, the peripheral dose and the treatment time achieved by FAAS plans were considerably superior for these patients. By contrast, the comparison of MU/fraction delivered between FAAS and PA plans reveals no significant difference in the large tumor group. What’s more, the treatment time of the FAAS plans was longer than that of PA plans in large tumor patients. Our results optimize the selection of different radiation techniques during lung SBRT treatment and can provide valuable information for clinical implement.

It was reported that the scattered radiation to patients was at first-order directly proportional to the MU [20], and the increase in peripheral dose could theoretically increase the risk of secondary malignancies [21, 22]. Since peripheral dose assessment can’t be easily calculated with a high degree of accuracy [18], we employed a setup of dynamic thorax phantom to measure the peripheral dose as a function of longitudinal distance from isocenter. Our data showed that the peripheral doses were obviously reduced along the longitudinal direction from the isocenter using the FAAS plans in the small tumor patients, suggesting its potential to lower the risk of secondary malignancies induced by radiotherapy.

In the present study, we used PA and FAAS techniques to accomplish SBRT treatment because full arcs rotation strategy appears to increase the disadvantageous dose to the contralateral lung. Lower dose to it was of concern because it was a risk factor to the incidence of RIP [7]. For SBRT treatment of lung cancer, the internal scatter of the patient also contributed to the contralateral lung dose. It may play a more significant role especially when the distance from the radiation central axis is usually less than 15 cm in lung cases. Since we found that the FAAS technique achieved significant MU reduction in the small tumor group, its contribution to the contralateral lung dose needs further investigation.

The treatment efficiency was evaluated via measuring the treatment time. The total treatment time includes three parts in RapidArc based lung SBRT: MU delivered time (equal to total MU divided by maximum dose rate), interval time between two arcs (about 5 seconds when the collimator was set to 30° and 330° rotations on the TrueBeam Linac), and the gantry rotation time (6°/s on TrueBeam) when the FAAS technique is used [6, 23, 24]. Our novel calculation model was demonstrated to be in an excellent agreement in treatment time with the measures ones. However, it is worthy of note that the calculated treatment time is noticeably shorter (ranged from 1–5 seconds) than the actual measured one. This is partially due to the shutter effect of Linac, where the unsaturated dose rate was generated at the beginning and the end of beam on time.

As illustrated in Fig 3, the FAAS plans achieved a particularly higher MU reduction in small tumor group. Both of the two techniques investigated possess the constant dose rate (1400MU/min) during the treatment process and the treatment time was therefore about thirty seconds shorter in average than that of the PA plans. Thirty seconds reduction of treatment time achieved by the FAAS plans is of utmost importance for SBRT [5]. Because 7 Gy dose could have been delivered within thirty seconds when FFF beams with the maximum dose rate (1400MU/min) are applied. On the other hand, the shorter treatment time generally introduces substantially superior patient stability and treatment accuracy, simultaneously reduces the likelihood of intrafractional baseline shifts in tumor position [25, 26]. Previous research regarding the target motion as a function of treatment time found the average time needed to maintain the target motion within 1 mm of translation or 1 degrees of rotational deviation was 5.9 min for thoracic tumors, implying an inevitable target motion beyond the threshold of 5.9 min [27]. As the mean delivery time in the small tumor group was 6.2±0.7 minutes for the PA plans and only 5.7±0.5 for the FAAS plans, we thought thirty seconds reduction in delivery time was critical for SBRT treatment of lung cancer. Although a reduction of delivery can be interesting in regards to tumor movement and patient positioning, its biological consequences are limited.

Delta4 and Portal dosimetry were also employed to verify the delivery accuracy. Table 3 showed that the mean γ value for both of the techniques was less than 0.5, indicating an excellent agreement between the calculated and measured dose. However, there are some limitations associated with the use of delta4 and portal dosimetry for plan validation. For small field, Christian et al [28] recommended the use of films for dose verification measurements in stereotactic radiosurgery due to its high resolution than other tools, and they found a good agreement with estimated data by Monte Carlo algorithm for films. Thus, the experimental verification with films for small field size, such as those implemented in lung SBRT, will be an interesting topic for our future studies.

## Conclusions

We have demonstrated that the FAAS delivery strategy is more beneficial for small tumor patients undergoing lung SBRT with FFF beams due to the reduction of MU, peripheral doses, and the improvement in treatment efficiency. In contrast, for large tumor patients, the PA delivery strategy is recommended because it required less treatment time with similar target coverage, OARs sparing and peripheral doses compared to that achieved by FAAS plan. It remains to be determined whether the treatment schemes investigated would improve the local control, limit the late toxicity, and ultimately prolong patient survival.
